# Vertical asymmetry in central color evaluation: How spatial position and scene structure modulate the perceived size of colored regions

**DOI:** 10.1167/jov.26.10.16

**Published:** 2026-10-14

**Authors:** Tomoya Okawa, Michael Makoto Martinsen, Hideki Tamura, Shigeki Nakauchi, Sérgio M. C. Nascimento, Tetsuto Minami

**Affiliations:** 1 Department of Computer Science and Engineering, Toyohashi University of Technology, Toyohashi, Japan; 2 Physics Center of Minho and Porto Universities (CF-UM-UP), University of Minho, Braga, Portugal

**Keywords:** color perception, visual asymmetry, central vision

## Abstract

Humans exhibit vertical asymmetry in color perception, with the lower visual field generally showing superior performance. We investigated whether this asymmetry extends to central vision by having participants judge the relative size of colored and grayscale regions within small, centrally presented stimuli (1.5° × 2.0°). Natural-scene images were partially desaturated so that either the upper or lower portion remained in color, and participants reported which region (colored or grayscale) appeared to be larger. To examine the role of scene structure, we also used pixel-shuffled versions of the same images, which preserved the vertical chromaticity distribution but removed scene recognizability. Two presentation durations were tested: 50 ms and 250 ms. With a brief presentation duration (50 ms), the colored region was overestimated in size relative to the grayscale region more strongly in the lower colored than in the upper colored condition for natural-scene images, *t*(19)  =  5.34,  *p*  <  0.001,  *d*  =  1.20 in Experiment 1; $F\left(1,14\right)\;=\;23.54,\;p\;<\;0.001,\;{\hat{\eta }}_{p}^{2}\;=\;0.627$ in Experiment 2-1, but this asymmetry was not observed for pixel-shuffled images, $F\left(1,14\right)=\;0.28,\;p\;=\;0.602,{\hat{\eta }}_{p}^{2}\;=\;0.020$. With a longer presentation duration (250 ms), the lower-half effect was significant across both natural-scene and pixel-shuffled conditions, $F\left(1,15\right)\;=\;9.85,\;p\;=\;0.007,\;{\hat{\eta }}_{p}^{2}\;=\;0.396$, with no significant interaction between stimulus type and color position, *F*(1, 15)  =  1.14,  *p*  =  0.303. These findings suggest that the lower visual space biases color evaluation even in central vision and that this bias emerges more readily when a natural-scene structure is available.

## Introduction

The information processing characteristics of the human visual system are not uniform across the visual field. In particular, a clear difference in information processing between the upper and lower visual fields has been reported. The performance field is the area within the visual field where visual information can be processed most efficiently, and it has been documented that the performance field in the lower visual field is larger than that in the upper visual field (Carrasco, Talgar, & Cameron, 2001; Corbett & Carrasco, 2011; Hanning, Himmelberg, & Carrasco, 2022). Furthermore, the object recognition accuracy achieved for moving targets during vehicle operation has been reported to be better in the lower visual field than in the upper visual field (Seya, Nakayasu, & Yagi, 2013). Asymmetry between the upper and lower visual fields has also been demonstrated in terms of color discrimination accuracy. Hue discrimination capabilities are superior in the lower visual field than in the upper visual field, although this result is believed to be strongly influenced by attention (Levine & McAnany, 2005). Additionally, anatomical asymmetry has been observed: the density of cone cells in the retinal area that projects to the lower visual field is greater than that in the area projecting to the upper visual field (Curcio et al., 1991).

These differences between the upper and lower visual fields are thought to be related to species-specific evolutionary factors. In humans, upper–lower visual field asymmetries have been discussed in relation to ecological demands, with the upper visual field often associated with global information processing and the lower visual field with local information processing (Previc, 1990). This difference is attributed to information in the lower visual field being more frequently used for hazard avoidance than information in the upper visual field is. In particular, color information processing, which is crucial for avoiding danger, is considered more developed in the lower visual field than in the upper visual field. In contrast, color discrimination in mice is better in the upper visual field than in the lower visual field, a pattern that has been discussed in relation to ecological pressures such as the detection of aerial predators (Qiu et al., 2021; Szatko et al., 2020; Wallace et al., 2013). Thus, the importance of the visual information contained in the upper and lower visual fields appears to vary by species, and this difference is reflected in their evolutionary histories.

However, these discussions have focused primarily on the peripheral vision, and the asymmetries of color information processing capabilities in the central visual field are not yet fully understood. The central visual field developed to acquire highly detailed visual information during human evolution, and mice do not possess this retinal area (Galindo-Romero et al., 2022). Although the central visual field of humans is known to have high spatial resolution, its color vision properties are not uniform and can be influenced by attention and psychological factors (Carrasco, Ling, & Read, 2004; Dzulkifli & Mustafar, 2013; Provis, Dubis, Maddess, & Carroll, 2013). One possibility is that the spatial positions “upper” and “lower” themselves carry a functional meaning shaped by evolutionary pressure. By this we mean that the lower visual field is ecologically linked to the ground and near space, where behaviorally important objects (e.g., food, obstacles, and footing) typically appear. If the positions “upper" and “lower" carry such significance, color perception may differ between them even within the central visual field, where anatomical asymmetries are small. Consistent with an interaction between visual field position and scene structure, responses in the early visual cortex depend on the visual field origin of natural-scene patches (Mannion, 2015).

Building on these considerations, the present study investigated whether and how the spatial positions “upper” and “lower” influence color evaluation in central vision. We used a size comparison task in which participants judged whether the colored or the grayscale portion of a centrally presented, partially desaturated natural scene appeared to be larger. This task provides an indirect but practical index of color-related processing, motivated by evidence that regions perceived as more important tend to be judged as larger (Anton-Erxleben, Henrich, & Treue, 2007) and that a larger colored area is generally perceived as more vibrant (Balas & Sinha, 2007; Loschky, McConkie, Yang, & Miller, 2005). To examine whether any such asymmetry depends on scene structure, we compared responses to natural-scene images with those to pixel-shuffled versions, in which horizontal pixel shuffling removed scene recognizability while preserving the vertical chromaticity distribution. Because this manipulation alters not only scene recognizability but also the lower-level image structure, we describe the comparison in neutral terms throughout (natural-scene vs. pixel-shuffled images) and consider its interpretation in the Discussion.

We conducted three experiments to address these questions. Experiment 1 used natural-scene images with a brief 50-ms presentation to characterize the baseline pattern under everyday-like conditions. In experiment 2-1, pixel-shuffled versions of the same images at the same brief duration were added to test whether a scene structure is needed for the bias to appear. Experiment 2-2 then used a longer duration (250 ms) to test how processing time interacts with scene structure. We reasoned that scene structure in natural scenes can be extracted rapidly and may engage the bias even at a brief duration, whereas pixel-shuffled images, which lack such structure, may require a longer viewing time for the bias to emerge.

## Experiment 1

Experiment 1 investigated whether there is a discrepancy between the perceived sizes of colored regions positioned in the upper and lower central visual fields on the basis of the assumption that highly important objects are perceived as larger (Anton-Erxleben et al., 2007). This experiment aimed to determine whether the spatial positions “upper” and “lower” affect color evaluation in central vision. The experiment used natural-scene images that were partially colored in one of seven vertical levels as visual stimuli, and the images were presented for a brief duration of 50 ms. The participants were asked to report whether the colored area or the noncolored area of the visual stimulus appeared to be larger.

### Participants

Twenty-one healthy students (5 females, 16 males; mean age, 22.00 ± 0.84 years) participated in the experiment. All the participants were students at Toyohashi University of Technology and reported having normal or corrected-to-normal vision and no self-reported color vision deficiencies. A formal color vision test (e.g., a chart-based screening test) was not administered. Instead, participants were screened using self-report questions about color-related difficulties and prior diagnosis. This screening procedure was kept consistent across the experiments. The required sample size was estimated to be 20 using G*Power (Faul, Erdfelder, Lang, & Buchner, 2007) for a paired two-tailed *t*-test (α = 0.050; 1 − β = 0.800; *d* = 0.675). Data collection continued until the target number of analyzable datasets was obtained. A dataset was treated as analyzable if the participant did not meet the exclusion criterion described below. The experiments were conducted with the approval of the Ethics Committee of Toyohashi University of Technology, and all the participants provided informed consent. The participants were not informed of the specific purpose or hypothesis of the study before their participation.

### Apparatus

The experiment was conducted in a dark room with a chin rest positioned 80 cm from the display. The visual stimuli were presented on an LCD monitor (VIEWPixx/3D, VPixx Technologies Inc., Saint-Bruno, Quebec, Canada) with a resolution of 1, 920  ×  1, 080 pixels and a refresh rate of 120 Hz. The color output of the monitor was measured using a spectroradiometer (SR-3AR; Topcon, Tokyo, Japan) to confirm the display calibration. The white point was set to D65, and the gamma values of each channel were (γ_*R*_,γ_*G*_,γ_*B*_) = (2.79,  2.72,  2.97). The visual stimuli were generated from the hyperspectral images and rendered for the calibrated display. The visual stimuli were presented using MATLAB R2023a and Psychtoolbox-3 (Brainard & Vision, 1997; Kleiner, Brainard, & Pelli, 2007; Pelli, 1997), running on a Windows 10 Home 22H2 system. The computer hardware used for the experiment included an Intel Core i7-9700K CPU @ 3.60 GHz, with 32 GB of RAM, and an NVIDIA Quadro P4000 GPU. Responses were collected using a controller with physical buttons instead of a keyboard.

### Stimuli

In the experiment, natural-scene images with partially colored upper or lower regions were used. Thirty natural-scene images were randomly selected from a natural-scene set (Foster & Reeves, 2022; Nascimento, Ferreira, & Foster, 2002) and generated using a D65 illuminant. To ensure presentation within the central visual field, the stimuli subtended a size of 1.5° vertically × 2.0° horizontally. The set of selected scene images is shown in Figure 1. There were two experimental conditions: the upper colored condition and the lower colored condition. Stimuli were created to correspond with these two conditions. The colored area of the stimuli varied across seven levels: full gray, 1/6, 2/6, 3/6, 4/6, 5/6, and full color (see Figure 2). In the upper colored condition, the image was partially colored starting from the top edge and extending downwards. On the other hand, in the lower colored condition, the image was partially colored starting from the bottom edge and extending upwards. Since the full-gray and full-color levels were identical in both the upper colored and lower colored conditions, their overlaps were excluded, resulting in a total of 7  ×  2 − 2 = 12 unique experimental conditions.

**Figure 1.**
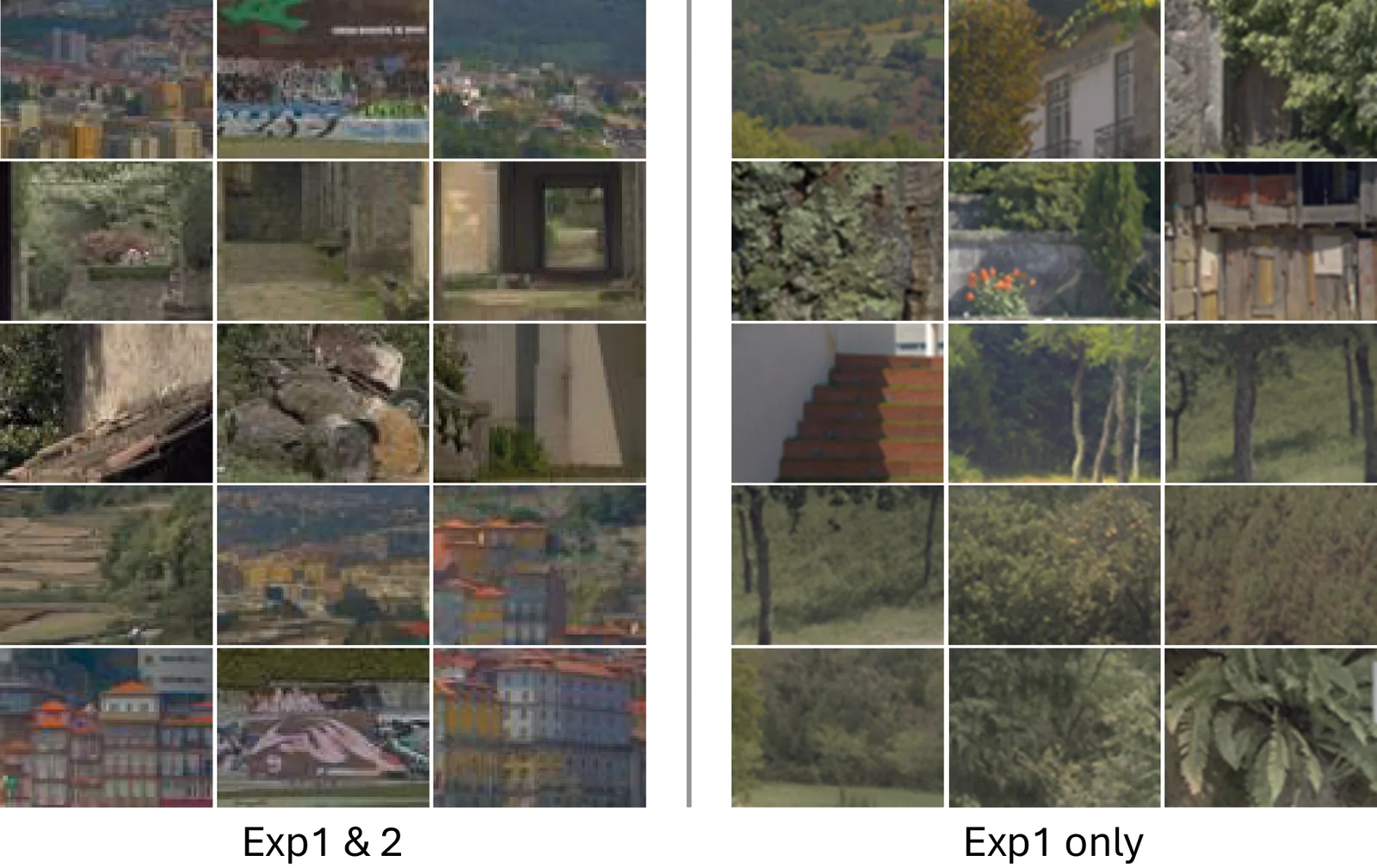
Natural-scene images used in the experiments. The 15 images on the left were used in both Experiment 1 and Experiment 2, whereas those on the right were used only in Experiment 1.

**Figure 2.**
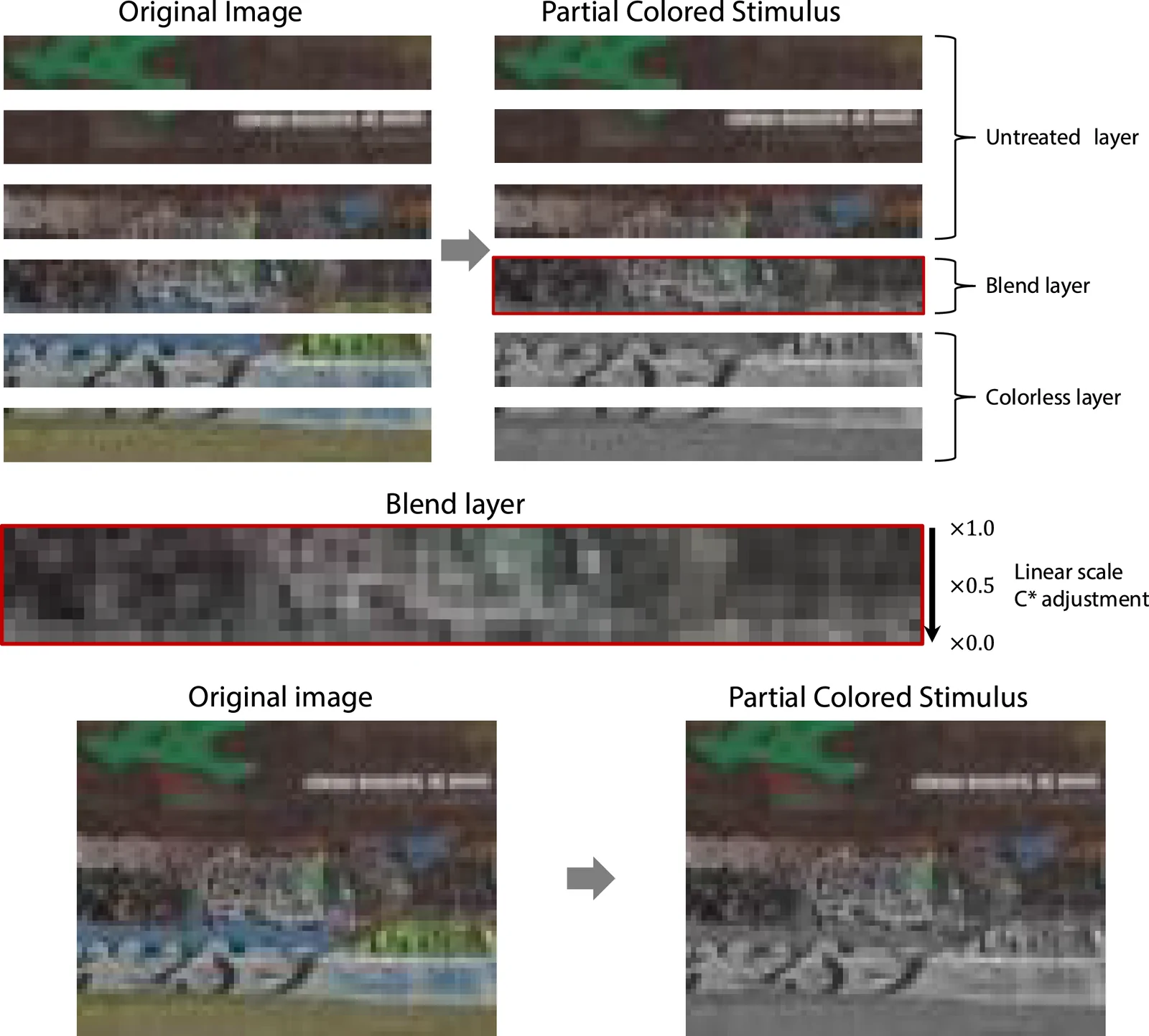
A partial desaturation operation was conducted on the upper 3/6 colorful section. The image was vertically divided into six sections, comprising three layers: an untreated layer, a blended layer, and a decolorization layer. The untreated layer underwent no desaturation; its size ratio corresponded with the experimental conditions. The blended layer, with a constant size ratio of 1/6, served to blur the boundary between the untreated and desaturation layers. The desaturation layer set the C* value in CIELAB to zero.

The stimuli were created by partially desaturating a full-color natural-scene image. The desaturation operation is shown in (Equations 1–4) below. Here, $C_{U_{i}}^{*}\left(x,y\right)$ is the C* distribution of the experimental stimulus with the upper *i*/6 region colored, and $C_{L_{i}}^{*}\left(x,y\right)$ is the C* distribution of the experimental stimulus with the lower *i*/6 region colored (where 1 ≤ *i* ≤ 5). C*(*x*, *y*) is the C* distribution of the original natural-scene image, and $F_{U_{i}}\left(y\right)$ and $F_{L_{i}}\left(y\right)$ are the filter functions for achieving partial desaturation in the upper and lower regions, respectively. Let *x* and *y* be the horizontal and vertical coordinates on the image plane, respectively, with the top-left corner as the origin and the bottom-right corner as the positive direction, and let *v* be the vertical size of the stimulus. The partial desaturation operation for the 3/6 upper colored condition is visually represented in Figure 2. This operation created 12 conditions × 30 natural-scene images = 360 unique partially colored images.

(1) $$\begin{array}{c} C_{U_{i}}^{*}\left(x,y\right)=C^{*}\left(x,y\right)\times F_{U_{i}}\left(y\right)\; \end{array}$$

(2) $$\begin{array}{c} C_{L_{i}}^{*}\left(x,y\right)=C^{*}\left(x,y\right)\times F_{L_{i}}\left(y\right)\; \end{array}$$

(3) $$\begin{array}{c} \;F_{U_{i}}\left(y\right)=\left\{\begin{array}{cc} 1 & \left(y<\frac{v\times i}{6}\right)\\ 1-\frac{6}{v}\left(y-\frac{v\times i}{6}\right) & \left(\frac{v\times i}{6}\leq y<\frac{v\times \left(i+1\right)}{6}\right)\\ 0 & \left(\frac{v\times \left(i+1\right)}{6}\leq y\right) \end{array}\right.\; \end{array}$$

(4) $$\begin{array}{c} \;F_{L_{i}}\left(y\right)=\left\{\begin{array}{cc} 0 & \left(y\leq \frac{v\times i}{6}\right)\\ \frac{6}{v}\left(y-\frac{v\times i}{6}\right) & \left(\frac{v\times i}{6}<y\leq \frac{v\times \left(i+1\right)}{6}\right)\\ 1 & \left(\frac{v\times \left(i+1\right)}{6}<y\right) \end{array}\right.\; \end{array}$$

Next, the mean L* value of the 360 partially colored images created in (Equations 1–4) was controlled using the operation shown in (Equation 5). Here, L* is the controlled L* distribution of an arbitrary partially colored image, L* is the L* distribution before the control scheme is implemented, $M_{L^{*}}$ is the overall mean L* value of the entire set of partially colored images, and $I_{L^{*}}$ is the mean L* value of the arbitrary partially colored image.

(5) $$\begin{array}{c} {L^{*}}^{{}^{'}}=L^{*}\times \frac{1}{M_{L^{*}}}I_{L^{*}}\; \end{array}$$

Finally, the mean C* of the corresponding upper and lower partial regions within the same natural-scene image was controlled using the operations shown in Equations (6–8).

(6) $$\begin{array}{c} C{}_{i}^{*}{}^{'}=\left\{\begin{array}{c} C^{*}\times \frac{M_{U_{i}}}{M_{L_{i}}}\left(M_{U_{i}}<M_{L_{i}}\right)\\ C^{*}\times \frac{M_{L_{i}}}{M_{U_{i}}}\left(M_{U_{i}}>M_{L_{i}}\right) \end{array}\right.\; \end{array}$$

(7) $$\begin{array}{c} M_{U_{i}}=\int_{U_{i}}C^{*}\left(S\right)dS\; \end{array}$$

(8) $$\begin{array}{c} M_{L_{i}}=\int_{L_{i}}C^{*}\left(S\right)dS\; \end{array}$$

### Procedure

The experiment followed the procedure illustrated in Figure 3. In each trial, an interstimulus interval and a fixation point were presented for 500 ms each to clearly demarcate the start of the trial and guide the gaze to the center. Afterwards, the experimental stimulus (subtending 1.5° × 2.0°) was presented for a brief duration of 50 ms in the center of the monitor (21.2° × 36.8°). This short duration was specifically chosen to minimize the influence of attention and to measure the responses that occurred during the early stages of visual processing (Liu, Yin, Guo, & Ye, 2024; Sperling, 1960). Immediately after the stimulus was presented, a noise mask was presented across the entire monitor for 1,000 ms. This noise mask was generated using a uniform random distribution, with L* values in the range 0 ≤ L* ≤ 25. After the noise mask was presented, the participants used a physical button to respond regarding whether they thought the colored domain or the grayscale domain of the presented stimulus appeared to be larger. This response was based on the hypothesis that objects with greater importance levels are perceived as larger (Anton-Erxleben et al., 2007). The experiment consisted of 30 repetitions for each of the 12 experimental conditions, resulting in a total of 360 main trials per participant.

**Figure 3.**
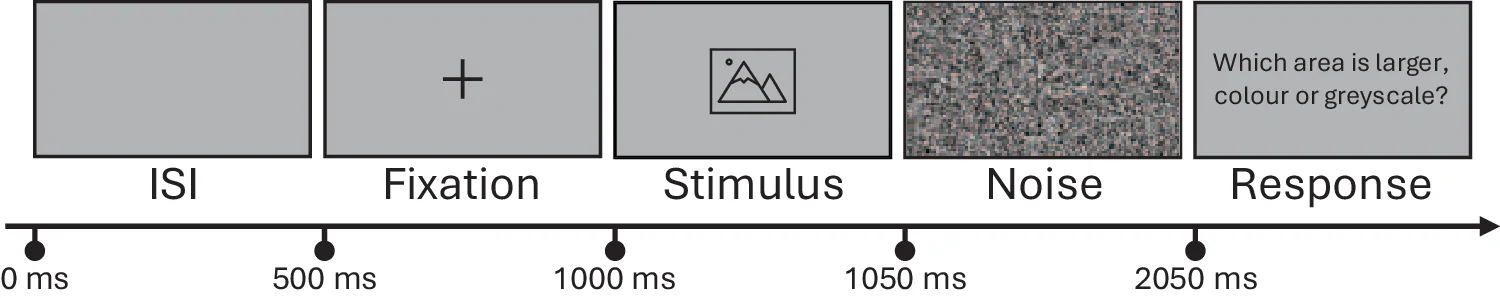
Procedure for Experiment 1. First, a 500-ms interstimulus interval (ISI) and a fixation point were presented. The experimental stimulus was subsequently presented for 50 ms, and this was immediately followed by a 1,000-ms noise pattern displayed across the entire screen. After the noise was presented, the participants responded according to the on-screen instructions, indicating whether they perceived the color domain or the grayscale domain to be larger.

Before the main trials were executed, 24 practice trials were conducted to familiarize the participants with the experimental procedure. Only during the practice trials was feedback provided regarding the correctness of the response. To enable this feedback, stimuli where the colored domain was exactly one-half of the visual stimulus (i.e., the 3/6 color condition) were not presented during the process. The stimuli used in the practice trials were a set of 24 randomly selected stimuli presented in a nonuniform distribution across the different conditions. Importantly, the practice stimuli were independent of the main stimuli and were not repeated in the main trials.

The participants were seated on a chair in a dark room with their chins placed on the chin rest, positioned in front of the monitor. Before starting the experiment, the participants were screened for self-reported color vision abnormalities using two questions: “Have you ever experienced any difficulty with colors in your daily life?” and “Have you ever been diagnosed with color blindness or a related condition by a doctor?” The participants were allowed to take a break freely in the dark room every 60 trials. The entire experiment took approximately 30 minutes to complete.

### Analysis

Before the analysis, a common exclusion criterion was established to screen out participants whose response patterns suggested that they had not reliably perceived the stimulus progression across the color-coverage levels, for example, because of inattention or blinking. Participants were excluded if their percentages of “colored” responses in either the upper colored or lower colored condition for the full color or 5/6 color levels fell below a threshold that was 20% lower than their response percentages for conditions with smaller colored areas. This rule was designed to remove data obtained from participants who frequently failed to correctly identify the stimuli. No participants met the exclusion criteria. However, the data obtained from one participant were corrupted because of a PC error and could not be included in the analysis. Consequently, 20 of the 21 participants were included in the final analysis set.

The primary analysis involved calculating the point of subjective equality (PSE) for the perceived size of the colored area under both the upper colored and lower colored conditions separately. The PSE represented the color occupation ratio at which the participants judged the colored and noncolored areas to be of equal size. The PSE values were calculated by fitting the percentage of “colored” responses against the color occupation ratio to an inverse cumulative normal distribution function using the glm function in R statistical software (R version 4.2.3; The R Foundation for Statistical Computing, Vienna, Austria). Following the calculation of the PSE values, a paired two-tailed *t*-test (Student's *t*-test) was conducted on the PSE values between the upper colored and lower colored conditions to test for a significant difference, using a significance level of 5%. The *t*-test function was used for this purpose. In addition, a Shapiro–Wilk test was performed on the differences between the samples (upper PSE minus lower PSE) to confirm the assumption of normality for the *t*-test.

### Results

The Shapiro–Wilk test conducted on the differences between the two conditions revealed no significant deviations from normality (*W* = 0.97; *p* = 0.706). The psychometric function curves for each individual participant are shown in Figure 4. A paired two-tailed *t*-test was performed on the PSE values of the upper colored condition (*M* = 0.53, *SD* = 0.11) and the lower colored condition (*M* = 0.45, *SD* = 0.11). The statistical results are shown in Figure 5. The results indicated that the PSE obtained for the lower colored condition was significantly lower than that observed for the upper colored condition, *M_D_* = 0.08, 95% confidence interval, 0.05 − 0.10, *t*(19) = 5.34, *p* < 0.001, *d* = 1.20. This statistically significant difference demonstrated asymmetry within the central visual field: relative to the grayscale region, the colored region was overestimated in size more strongly when it appeared in the lower field than when it appeared in the upper field.

**Figure 4.**
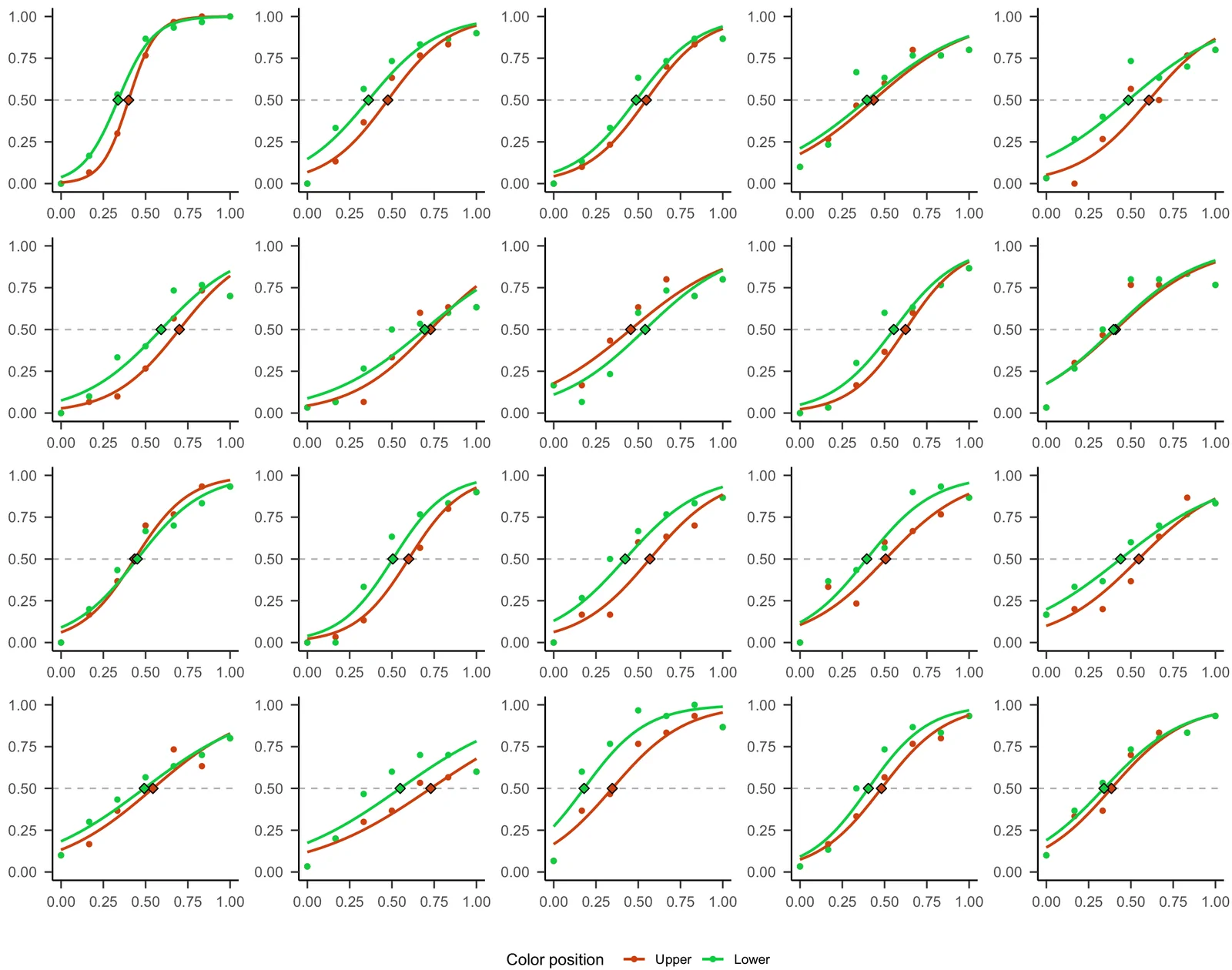
Psychometric functions for Experiment 1. Each panel shows one participant (*N* = 20). The points are the observed proportion of trials judged as “colored larger,” the curve is the fitted psychometric function, and the diamonds indicate the participants’ points of subjective equality, where the curve crosses 0.5 (dashed line). Red and green represent the upper colored and lower colored conditions, respectively. In all panels, the *x*-axis is the colored-area ratio (0 to 1), and the *y*-axis is the percentage of trials on which the colored region was judged to be larger than the grayscale region.

**Figure 5.**
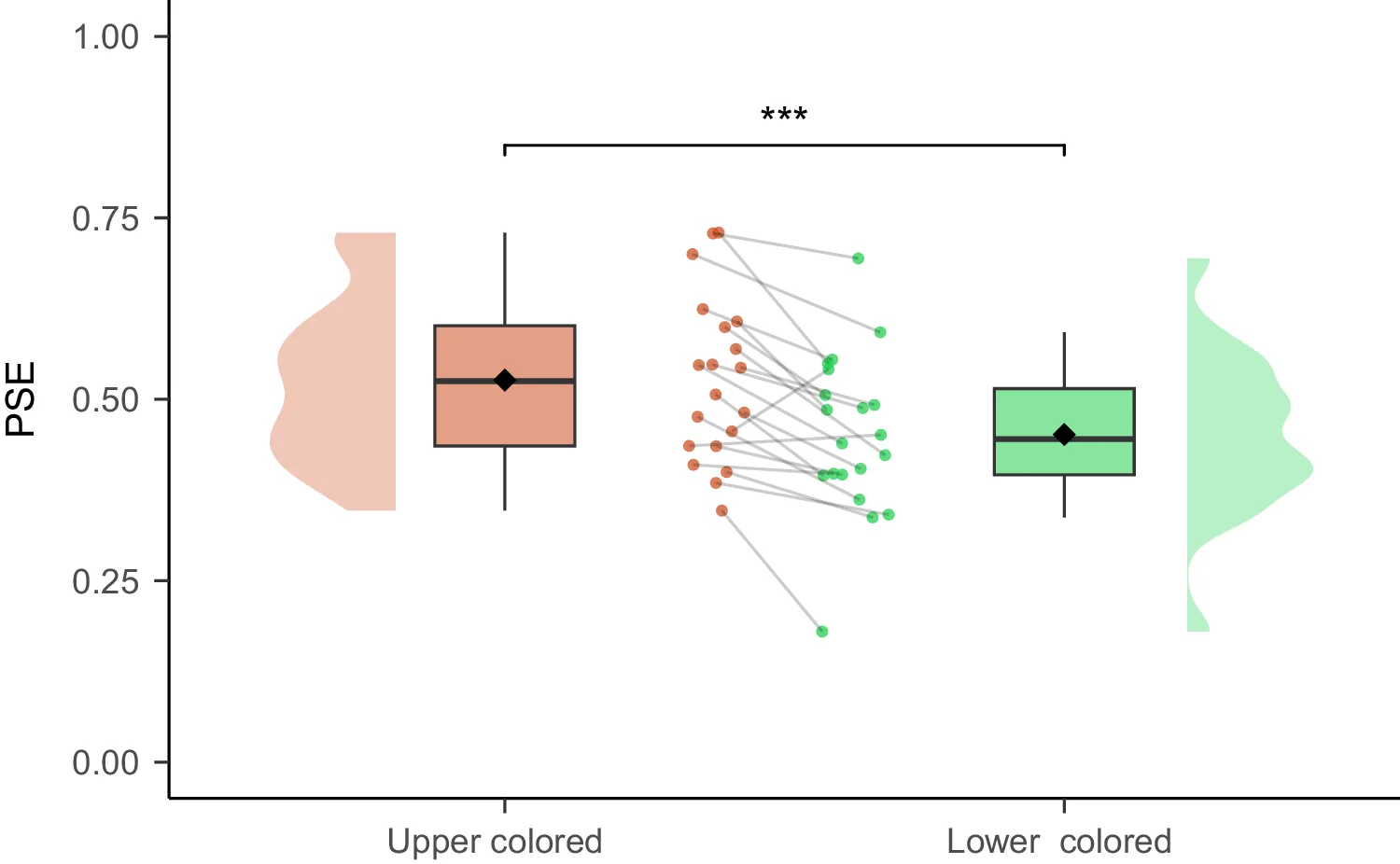
The results of Experiment 1 (***p < 0.001). The vertical axis represents the points of subjective equality (PSE) calculated for the perceived sizes of the colored and grayscale domains within the experimental stimulus, the horizontal axis denotes the experimental condition, and the scatter plot shows each participant's data.

### Discussion

The results from Experiment 1 demonstrated that the PSE was significantly lower in the lower colored condition than in the upper colored condition. The present task measured the relative size of the colored and grayscale regions, so this difference could, in principle, reflect an enlargement of the colored region in the lower field, a shrinkage of the grayscale region in the upper field, or both. Prior work has shown that a physically larger colored area tends to appear to be more vibrant (Balas & Sinha, 2007; Loschky et al., 2005); if the colored region in the lower field was overestimated in size, one interpretation is that chromatic information is processed in a way that produces a larger, and possibly more vibrant, percept, even within central vision. This pattern is consistent with the lower-field bias in color processing previously reported for the effective visual field (Levine & McAnany, 2005). These studies concern physically larger areas appearing to be more vibrant, not the reverse, so our link from size to color appearance is indirect and was not measured directly. The same result could also reflect a lower-field disadvantage: if chromatic information is localized less precisely there, the colored region might appear to spread and so be judged as larger. Distinguishing these accounts would require direct measures of color appearance, which the present task did not provide.

However, the vertical asymmetry in the effective visual field is often explained by physical factors, such as the distribution density of cone cells (Curcio et al., 1991). Given the relatively uniform cone cell distribution in the fovea, the asymmetry observed in Experiment 1 is unlikely to be explained by cone density alone. This does not, however, rule out other physiological factors, such as asymmetries in retinal ganglion cells or cortical representation; the result is nonetheless consistent with an additional contribution from higher-level factors, one possibility being a functional meaning assigned to the spatial information term “lower.” Marigold and Patla (2008) reported that humans prioritize visual information in the lower visual field over that in the upper visual field during locomotion (walking) (Marigold & Patla, 2008), demonstrating vertical asymmetry from the perspective of visual information importance. This finding is consistent with the possibility that the spatial information term “lower" has a functional meaning that contributes to the observed lower-field bias, even in the central vision; however, because the present task compared only the upper and lower positions, it cannot by itself separate this account from the physical fact of lower-field presentation, an issue we return to in Experiment 2.

A separate question is whether this lower-field bias is specific to natural-scene stimuli or reflects a more general property of visual processing. Although vertical asymmetry in terms of color information processing was observed, it is possible that this tendency is specific to natural-scene stimuli. Tesileanu, Piasini, and Balasubramanian (2022) discussed the possibility of different encoding time courses for simple visual figures versus stimuli containing semantic content during neural encoding, suggesting that the encoding time might also differ on the basis of feature integration theory (Treisman & Gelade, 1980). If the vertical asymmetry observed in this experiment was a result of the neural encoding of color information, the reactions to visual stimuli other than natural scenes should be investigated. Therefore, in Experiment 2, the influence of scene structure on color evaluation is investigated by examining the interactions between natural-scene images and pixel-shuffled images. Pixel shuffling removes scene recognizability while preserving the vertical chromaticity distribution.

## Experiment 2

Experiment 1 revealed a lower-field bias in the evaluation of colored regions using natural-scene images. However, these findings may be specific to stimuli in which the scene structure is intact. Experiment 2 therefore introduced pixel-shuffled images, which removed scene recognizability while preserving the vertical chromaticity distribution, to examine whether the observed bias depended on scene structure.

Experiment 2 comprised two subexperiments. Experiment 2-1 used the same brief presentation duration as that used in Experiment 1 (50 ms) to examine whether the lower-field bias differed between natural-scene and pixel-shuffled images. Experiment 2-2 used a longer presentation duration (250 ms) to examine whether the results of Experiment 2-1 were modulated by the time available for visual processing. Although the two subexperiments used independent groups of participants, they shared the same apparatus, stimuli, and experimental design, differing only in the presentation duration.

### Experiment 2-1

#### Participants

Twenty-one students participated in Experiment 2-1 (6 females, 15 males; mean age, 21.00 ± 2.26 years). The initial sample size was estimated to be 16 using PANGEA (Westfall, 2015) (for a 2  ×  2 within-subjects design, with *d* = 0.45, *rep* = 105). Data collection was intended to continue until at least 16 analyzable datasets were obtained, where analyzability was determined on the basis of the common exclusion criterion described elsewhere in this article. However, owing to time constraints, the experiment was concluded after data were collected from 21 participants. The final number of analyzable datasets included in the analysis was 15. None of the participants in this experiment had participated in the previous experiments.

#### Apparatus

The computer used for presenting the visual stimuli was operated under a newer Windows system (Windows 11 Home 23H2). All other experimental settings remained the same as those described for Experiment 1. These were the LCD monitor (VIEWPixx/3D), the viewing distance (80 cm), the chin rest, the dark room setup, and the use of MATLAB R2023a and Psychtoolbox-3.

#### Stimuli

For Experiment 2, the experimental stimuli used comprised those created for Experiment 1, supplemented by versions that were subjected to pixel shuffling (applied only in the horizontal direction). Horizontal-only pixel shuffling was performed to disrupt the scene structure of each image while simultaneously preserving its original vertical chromaticity distribution (the vertical change in color saturation). This pixel-shuffling operation is illustrated in Figure 6. To maintain a total experimental duration that was comparable with that of Experiment 1, the number of natural-scene images used in Experiment 2 was halved from 30 in the original set to 15. These 15 natural-scene images were intentionally selected to exclude images with overly similar compositions. The specific natural-scene images used in Experiment 2 are shown in Figure 1.

**Figure 6.**
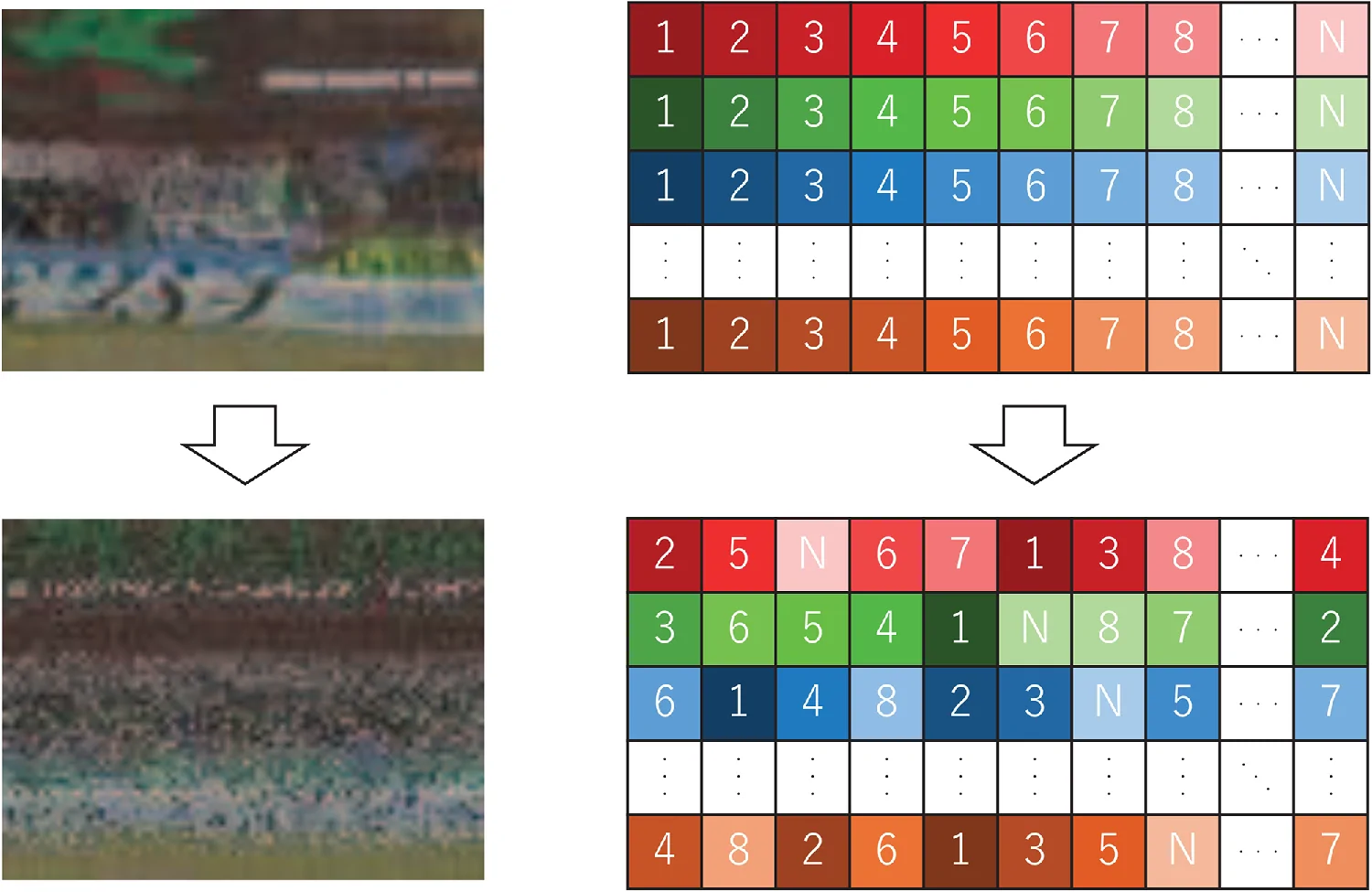
Pixel shuffling operation applied to the experimental stimuli. Pixel shuffling was applied only in the horizontal dimension, preserving the average chromaticity distribution in the vertical direction.

#### Procedure

The procedure for a single trial in Experiment 2 was identical to that in Experiment 1. Experiment 2 was divided into two main parts: a first session and a second session. Stimuli that were pixel shuffled and stimuli that were not pixel shuffled (natural scene) were presented in separate sessions. The order of these sessions (i.e., whether the natural-scene images were presented first or the pixel-shuffled images were presented first) was counterbalanced across the participants to control for order-related effects. The participants were required to take a 3-minute mandatory break between the first and second sessions. As in Experiment 1, practice trials were conducted before the main experiment began. In Experiment 2, both the natural-scene stimuli and the pixel-shuffled stimuli were presented during these practice trials to ensure that the participants understood the task across both stimulus types.

#### Analysis

Using the same exclusion criteria as those used in Experiment 1, participants were removed from the analysis if their percentages of “colored” responses produced under the full color or 5/6 color conditions for either the upper or lower colored condition fell below a threshold that was 20% lower than their response percentages for conditions with smaller colored areas. This procedure led to the exclusion of six participants. Consequently, 15 of the 21 participants were included in the final analysis set.

The analysis focused on the PSE determined for the perceived size of the colored area under each condition. A two-way repeated-measures analysis of variance (ANOVA) was conducted on the PSE values, with a significance level of 5%. The two factors were the image-type condition (two levels: a natural-scene condition and a pixel-shuffled condition) and the color position condition (two levels: an upper colored condition and a lower colored condition). To confirm the prerequisites for the ANOVA, a Shapiro–Wilk test was performed on the residuals of the overall sample to check for the assumption of normality.

To evaluate the robustness of the results against the relatively high exclusion rate, we additionally conducted a sensitivity analysis using a more lenient exclusion criterion that retained approximately 80% of the sample (*N*  =  17). Under this alternative criterion, the interaction between the image-type condition and color position remained significant, $F\left(1,16\right)\;=\;6.17,\;p\;=\;0.024,\;{\hat{\eta }}_{p}^{2}\;=\;0.278$, as did the main effect of color position, $F\left(1,16\right)\;=\;5.49,\;p\;=\;0.032,\;{\hat{\eta }}_{p}^{2}\;=\;0.255$, whereas the main effect of the image-type condition remained nonsignificant, *F*(1, 16)  =  0.52,  *p*  =  0.481. The simple main effect of color position was significant within the natural-scene condition, $F\left(1,16\right)\;=\;15.68,\;p\;=\;0.001,\;{\hat{\eta }}_{p}^{2}\;=\;0.495$, but not within the pixel-shuffled condition, $F\left(1,16\right)\;=\;0.15,\;p\;=\;0.701,\;{\hat{\eta }}_{p}^{2}\;=\;0.009$, reproducing the pattern obtained with the primary criterion. This finding indicates that the principal findings of Experiment 2-1 are robust to the choice of the exclusion threshold.

#### Results

The Shapiro–Wilk test performed on the residuals revealed no significant deviations from normality (*W* = 0.97; *p* = 0.111). The psychometric function curves produced for individual participants across all conditions are shown in Figure 7. A two-way repeated-measures ANOVA was conducted on the PSE values with the factors of the image-type condition (natural scene/pixel shuffled) and the color position condition (upper/lower). The statistical results are shown in Figure 8, and the descriptive statistics for each condition are summarized in Table 1. The analysis revealed a significant interaction between the image-type condition and the color position condition, *F*(1, 14) = 10.54, *p* = 0.006, ${\hat{\eta }}_{p}^{2}=0.430$. The main effect of the image-type condition was not significant, *F*(1, 14) = 0.00, *p* > 0.999, ${\hat{\eta }}_{p}^{2}=0.000$, and the main effect of the color position condition was significant, *F*(1, 14) = 9.96, *p* = 0.007, ${\hat{\eta }}_{p}^{2}=0.416$. A subsequent analysis of the simple main effects confirmed that the effect of the color position was significant within the natural-scene condition, *F*(1, 14) = 23.54, *p* < 0.001, ${\hat{\eta }}_{p}^{2}=0.627$, but was either not significant or substantially weaker in the pixel-shuffled condition, *F*(1, 14) = 0.28, *p* = 0.602, ${\hat{\eta }}_{p}^{2}=0.020$. These findings indicate that, relative to the grayscale region, the colored region tended to be more strongly overestimated in size when it appeared in the lower field (the main effect of color position was significant) and that this tendency was more pronounced for natural-scene images (the interaction and main effect were significant)

**Figure 7.**
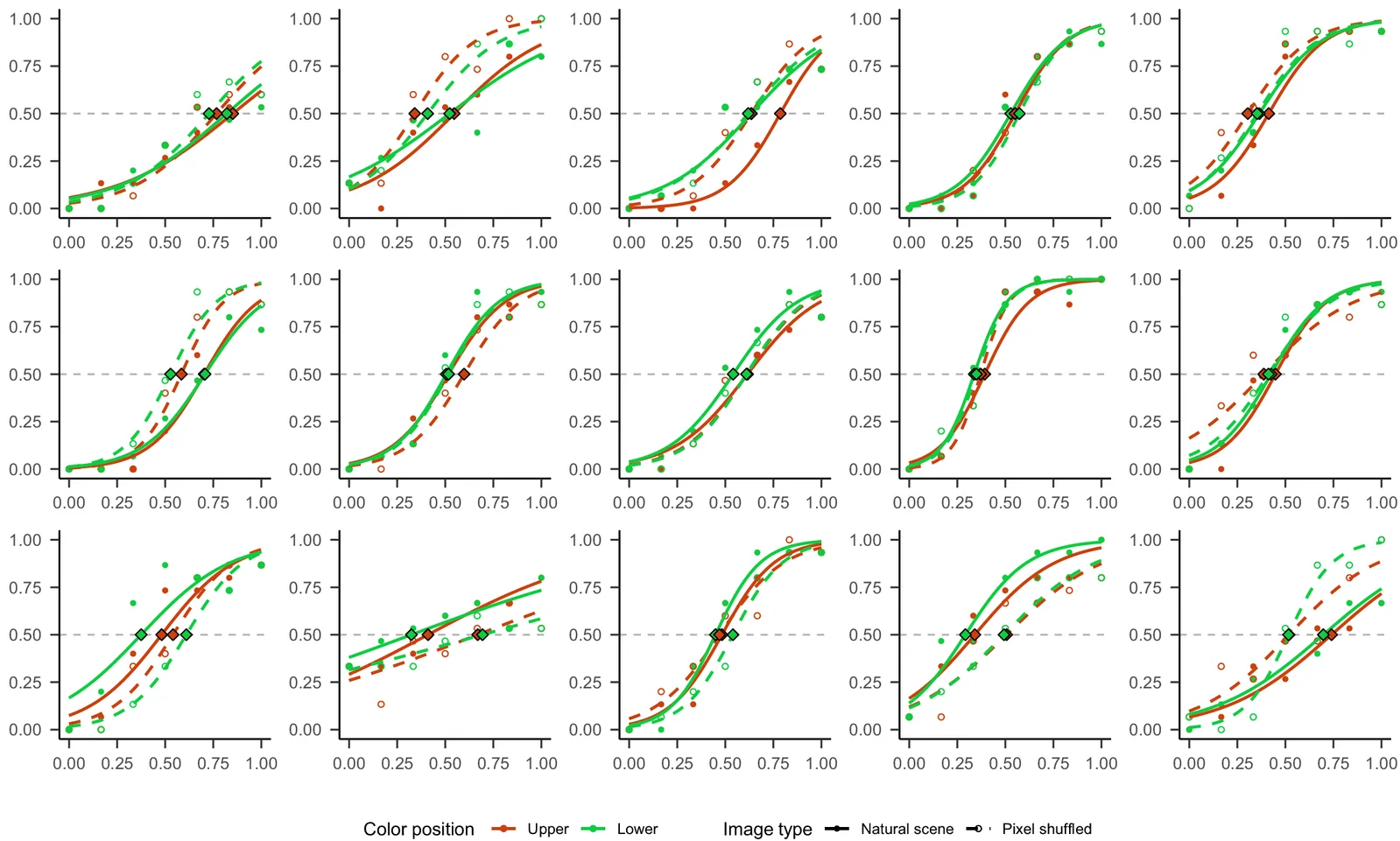
Psychometric functions for Experiment 2-1. Each panel represents one participant (*N* = 15). The points are the observed proportions, the curves are the fitted psychometric functions, and the diamonds indicate the participants’ points of subjective equality, where each curve crosses 0.5 (dashed line). Red and green show the upper and lower colored conditions, respectively; solid and dashed lines show the natural-scene and pixel-shuffled images, respectively. The axes are as in Figure 4.

**Figure 8.**
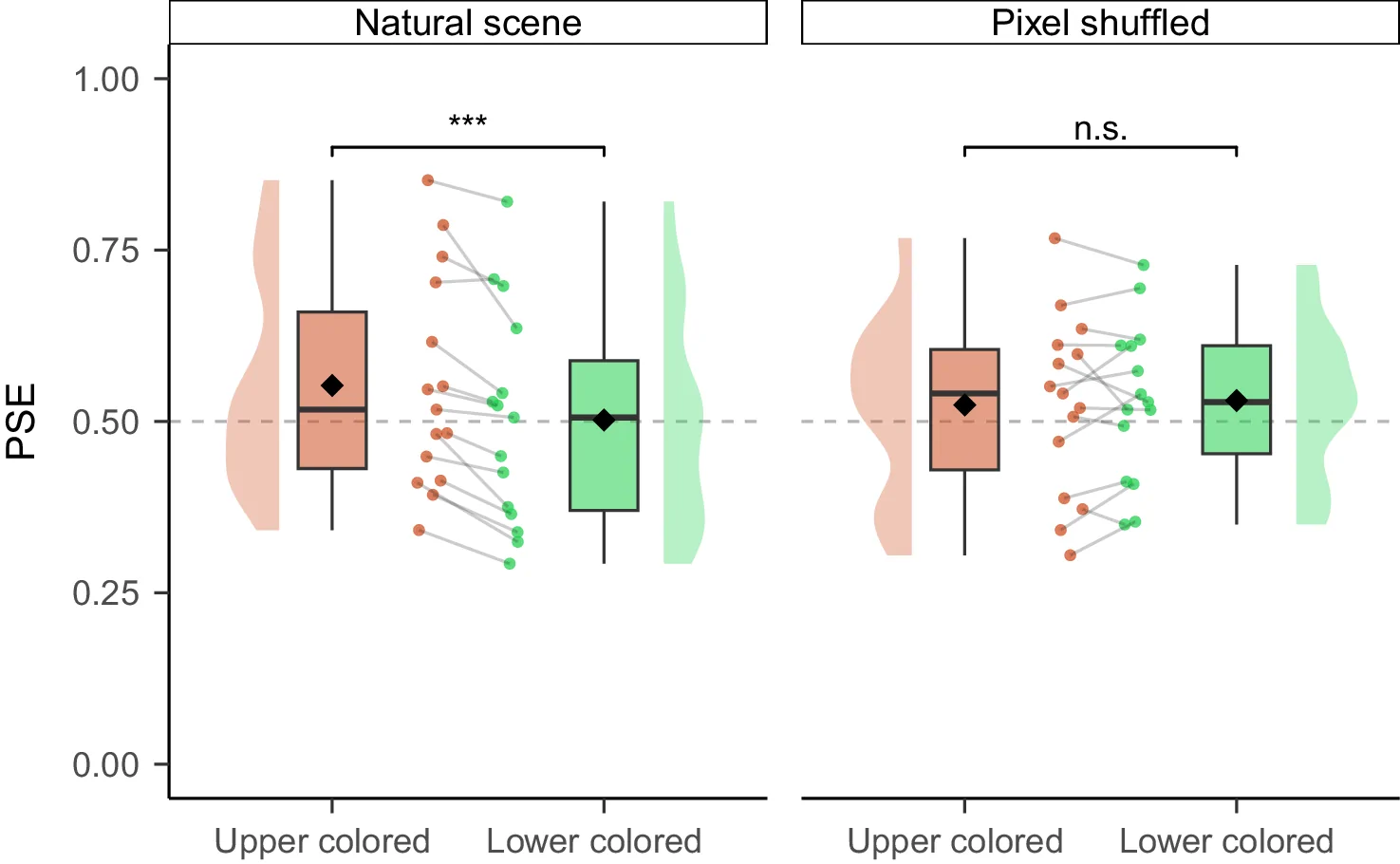
The results of Experiment 2-1 (***p < 0.001; *n*.*s*.,  not significant). The vertical axis represents the points of subjective equality (PSE) determined for the perceived sizes of the color and grayscale domains within the experimental stimulus, the horizontal axis denotes the experimental condition, and the scatter plot shows each participant's data.

**Table 1.** Descriptive statistics of the points of subjective equality values under each condition in Experiment 2-1.

Image-type condition	Color position condition	Mean	Standard deviation
Natural scene	Upper	0.55	0.16
Natural scene	Lower	0.50	0.16
Pixel shuffled	Upper	0.52	0.13
Pixel shuffled	Lower	0.53	0.11

#### Discussion

Experiment 2-1 revealed that the lower-field bias was stronger for natural-scene images than for pixel-shuffled images. This finding indicates that scene structure, which is disrupted by pixel shuffling, contributes to bias under brief presentation (Olshausen & Field, 1996; Tesileanu et al., 2022; Treisman & Gelade, 1980). We consider what this manipulation did and did not isolate in the General discussion. The absence of significant asymmetry for pixel-shuffled images at this brief duration raises the question of whether the bias is genuinely absent for such stimuli or whether it simply requires more processing time. Natural scenes contain scene structures that can be extracted rapidly (Oliva, 2005; Potter, 1976), which may allow the bias to be engaged within a brief 50-ms presentation, whereas pixel-shuffled images lack this structure and may need a longer viewing time. To test this, Experiment 2-2 used a longer presentation duration of 250 ms, which is generally considered sufficient for the stable extraction of features from natural scenes (Oliva, 2005; Potter, 1976).

### Experiment 2-2

In Experiment 2-1, the lower-field bias was significant for natural-scene images but not for pixel-shuffled images at a brief duration of 50 ms. One interpretation is that the scene structure of natural scenes is extracted rapidly and can engage the bias within a brief presentation, whereas pixel-shuffled images, which lack this structure, require a longer viewing time for the bias to emerge. Experiment 2-2 tested this theory using a longer duration of 250 ms, which is generally considered sufficient for the stable extraction of features from natural scenes (Oliva, 2005; Potter, 1976). If this interpretation is correct, the bias should also appear for the pixel-shuffled images at the longer durations.

#### Participants

Sixteen students participated in Experiment 2-2 (16 males; mean age, 20.69 ± 0.70 years). The initial sample size was estimated to be 16 using PANGEA (Westfall, 2015) (for a 2  ×  2 within-subjects design, with *d* = 0.45, *rep* = 105). The plan was to continue the data collection process until a minimum of 16 valid datasets were acquired. None of the participants in this experiment had participated in the previous experiments.

#### Apparatus and stimuli

The experimental apparatus, environment, and visual stimuli used in Experiment 2-2 were entirely identical to those used in Experiment 2-1.

#### Procedure

The only intended change in Experiment 2-2, as outlined in the Introduction, was the duration of the stimulus presentation period.

#### Analysis

Following the same exclusion criteria as those used in Experiment 1, the participants were removed from the analysis if their percentages of “colored” responses produced in the full-color or 5/6 color conditions for either the upper or lower colored condition fell below a threshold that was 20% lower than their response percentages obtained for conditions with smaller colored areas. This procedure did not lead to the exclusion of any participants. Consequently, no participants were excluded from the final analysis set.

The analysis focused on the PSEs calculated for the perceived size of the colored area under each condition. A two-way repeated-measures ANOVA was conducted on the PSE values, with a significance level of 5%. The two factors were the image-type condition and color position condition. To confirm the prerequisites for implementing the ANOVA, a Shapiro–Wilk test was performed on the residuals of the overall sample to check for the assumption of normality.

#### Results

The Shapiro–Wilk test performed on the residuals revealed no significant deviations from normality (*W* = 0.97; *p* = 0.195). The psychometric function curves produced for individual participants across all conditions are shown in Figure 9. A two-way repeated-measures ANOVA was conducted on the PSE values with the factors of the image-type condition (natural scene/pixel shuffled) and the color position condition (upper/lower). The statistical results are shown in Figure 10, and the descriptive statistics for each condition are summarized in Table 2. The analysis revealed no significant interactions between the image-type condition and the color position condition, *F*(1, 15) = 1.14, *p* = 0.303, ${\hat{\eta }}_{p}^{2}=0.071$. The main effect of the image-type condition was not significant, *F*(1, 15) = 1.00, *p* = 0.334, ${\hat{\eta }}_{p}^{2}=0.062$, and the main effect of the color position condition was significant, *F*(1, 15) = 9.85, *p* = 0.007, ${\hat{\eta }}_{p}^{2}=0.396$. These findings indicate that, compared with the findings of Experiment 2-1, allowing sufficient time for visual information processing renders the lower-field bias more pronounced not only under the natural-scene condition but also under the pixel-shuffled condition.

**Figure 9.**
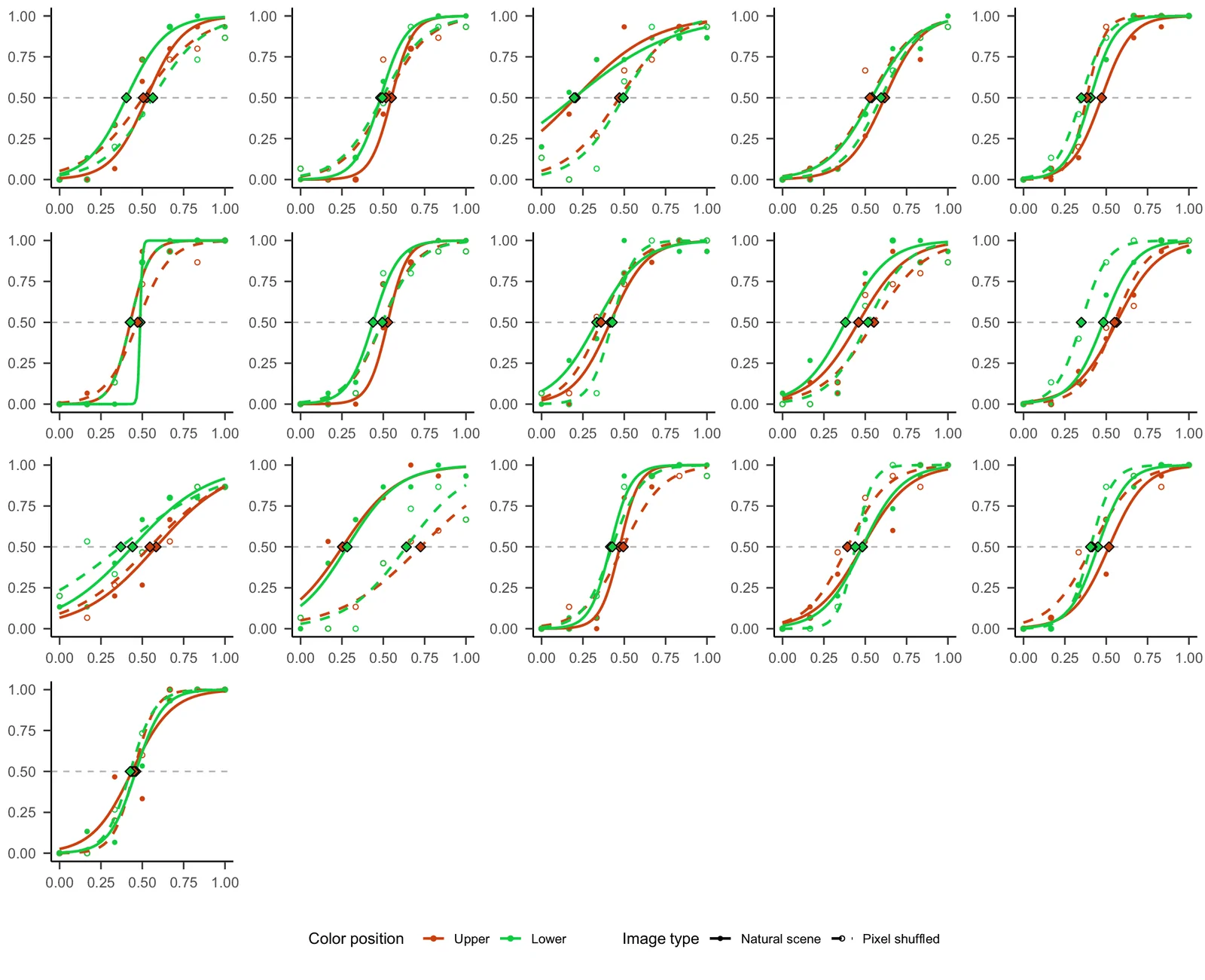
Psychometric functions for Experiment 2-2. Each panel shows one participant (*N* = 16). The points are the observed proportions, the curves are the fitted psychometric functions, and the diamonds indicate the participants’ points of subjective equality, where each curve crosses 0.5 (dashed line). Red and green show the upper and lower colored conditions, respectively; solid and dashed lines show the natural-scene and pixel-shuffled images, respectively. The axes are as in Figure 4.

**Figure 10.**
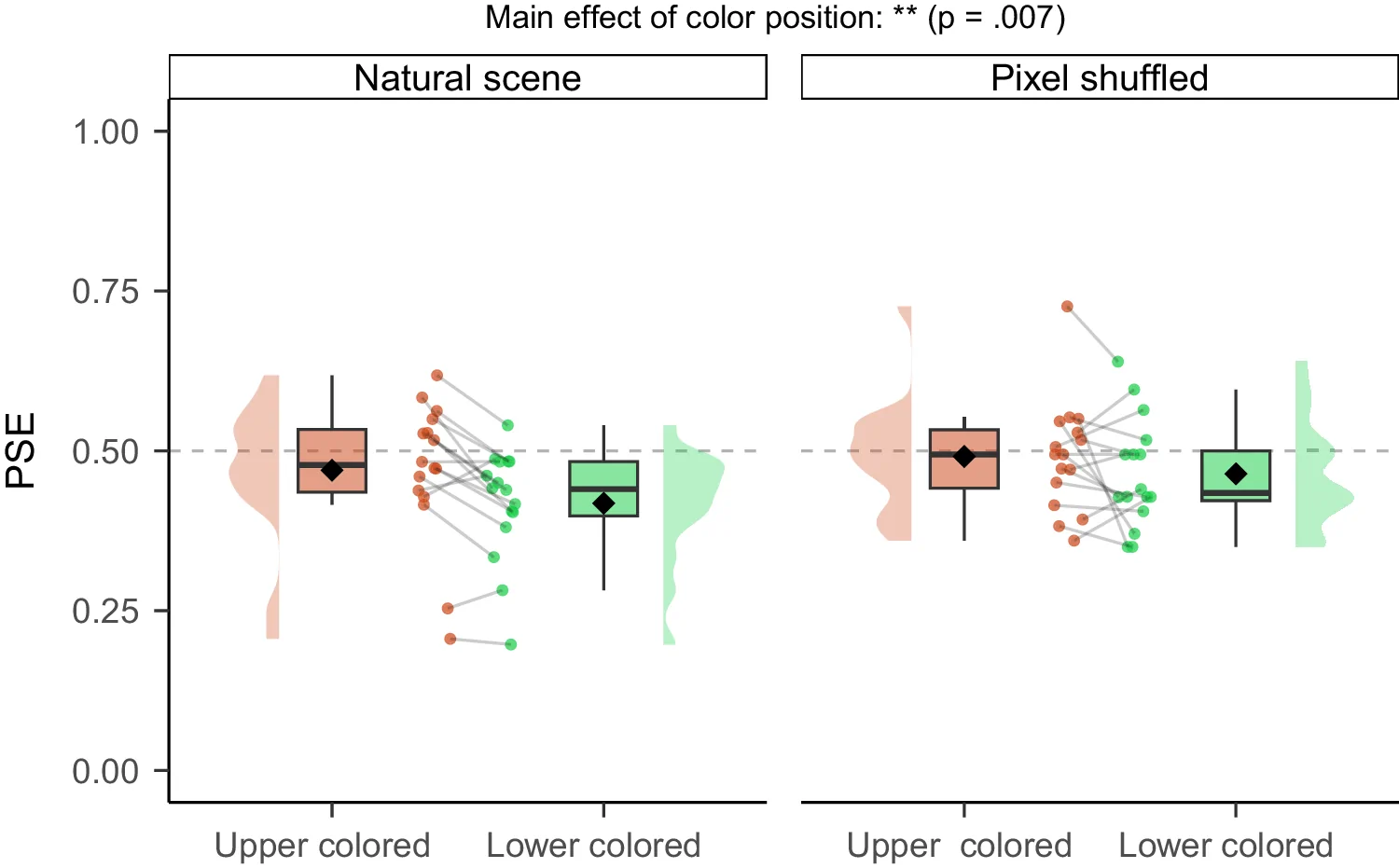
The results of Experiment 2-2 (**p < 0.01). The vertical axis represents the points of subjective equality (PSE) produced for the perceived size of the color and grayscale domains within the experimental stimulus, the horizontal axis denotes the experimental condition, and the scatter plot shows each participant's data.

**Table 2.** Descriptive statistics of the points of subjective equality values under each condition in Experiment 2-2.

Image-type condition	Color position condition	Mean	Standard deviation
Natural scene	Upper	0.47	0.11
Natural scene	Lower	0.42	0.09
Pixel shuffled	Upper	0.49	0.09
Pixel shuffled	Lower	0.46	0.08

#### Discussion

These results revealed that when the stimulus presentation time was 250 ms, the interaction observed in Experiment 2-1 was no longer significant, and only the main effect of color position remained significant. The lower-field bias was thus present for both natural-scene and pixel-shuffled images and was not restricted to the natural-scene condition as in Experiment 2-1. This pattern is consistent with the possibility that the lower-field bias is a general tendency that can be engaged by either stimulus type when sufficient processing time is available, whereas at a brief 50-ms duration, it was engaged only for natural-scene images, perhaps because their scene structure can be extracted more rapidly (Oliva, 2005; Potter, 1976). These observations broadly align with reports that spatial processing contributes to lower-field advantages (Genzano, Di Nocera, & Ferlazzo, 2001), although the present design does not directly test feedforward versus recurrent contributions. A more detailed discussion of this account is provided in the General discussion.

## General discussion

Across the three experiments, we observed a consistent pattern: relative to the grayscale region, the colored region was overestimated in size more strongly when it was positioned in the lower one-half of the stimulus than when it was positioned in the upper one-half. This lower-field bias was present for natural-scene images at both brief (50 ms) and longer (250 ms) presentation durations, whereas for pixel-shuffled images, it emerged only at longer durations. These results suggest that the lower-field bias is generally enhanced by scene structure and modulated by the time available for visual processing.

The experimental results demonstrate that the lower-field advantage previously established for the effective visual field is preserved for color-information processing even when it is limited to central vision. The vertical asymmetry exhibited by color vision is attributed to both low-level factors (e.g., asymmetric cone cell densities in the upper/lower retina) and high-level factors (e.g., attention and spatial processing). Given that the fovea (the core of central vision) has an extremely high and relatively uniform cone cell density for sharp vision (Provis et al., 2013), the asymmetry observed in our central visual field task cannot be fully explained by the density differences alone. This does not, however, exclude other physiological asymmetries (e.g., in retinal ganglion cells or cortical representation), which we cannot rule out with the present data. It is, nonetheless, consistent with an additional contribution from higher-level factors. One possibility is that the spatial information term "lower" carries an implicit functional meaning that influences color evaluation, although the present data do not directly establish this link. The finding that the colored region is overestimated relative to the grayscale region more strongly in the lower field is consistent with the idea that humans judge visual information in the lower field as more important, a concept that is supported by studies on safety awareness during locomotion (Marigold & Patla, 2008). If this interpretation is correct, such a bias (possibly related to the functional significance of lower visual space) may contribute to the observed lower-field effect in color evaluations, alongside or beyond low-level factors. The clearest evidence in our study for such a contribution comes from Experiment 2. Here, the bias changed when the scene structure was disrupted, even when the vertical chromaticity distribution was preserved, which a purely position-based account would not predict.

The bias was particularly pronounced for natural-scene images at the brief (50 ms) duration, which is consistent with the possibility that the spatial layout of natural scenes, such as object placement and depth, engages the lower-field bias more rapidly than pixel-shuffled stimuli do.

The time-dependent differences between Experiment 2-1 (50 ms) and Experiment 2-2 (250 ms) may be loosely related to the interaction between feedforward processing (fast category recognition) and recurrent processing (perceptual enhancement) in the ventral pathway (Lamme & Roelfsema, 2000; Riesenhuber & Poggio, 1999; Serre, Oliva, & Poggio, 2007; Thorpe, Fize, & Marlot, 1996). At 50 ms, rapid feedforward processing may be sufficient to engage the lower-field bias for natural-scene images, which provide scene structures that can be extracted quickly. The absence of the bias for pixel-shuffled images at the same duration, and its emergence at 250 ms, is consistent with, although not direct evidence for, the role of additional recurrent processing when such a structure is unavailable. Because the present design did not directly measure feedforward versus recurrent contributions, this account should be taken as a plausible framing rather than an established mechanism.

These higher-level interpretations, however, depend on the assumption that the difference between natural-scene and pixel-shuffled stimuli can be attributed to semantic content. By “semantic content” we mean a high-level, interpretive property of an image (its recognizable objects, surfaces, and layout), which is supported by a low-level structure such as spatial coherence and the distribution of oriented contrasts rather than being separate from it. In this view, spatial coherence and the distribution of oriented contrasts are not themselves semantic content; they are part of the low-level basis from which it arises. It is, therefore, important to consider the limitations of pixel shuffling manipulation and the potential role of low-level stimulus characteristics. In the present study, pixel shuffling was applied only in the horizontal direction to remove the recognizability of each scene while preserving the vertical chromaticity distribution. However, this operation altered these low-level properties (including spatial coherence, the distribution of oriented contrasts, and local texture within horizontal bands) and, with them, the recognizability that they support. Therefore, the difference between the natural-scene and pixel-shuffled conditions cannot be attributed solely to the presence or absence of semantic content; it may also reflect differences in spatial structure that engage different levels of visual processing. We, therefore, regard semantic content as one interpretation of this difference rather than as a property that our manipulation isolated.

Furthermore, natural-scene images inherently contain vertical asymmetries in their low-level statistics. The upper regions of outdoor scenes typically have a greater luminance (corresponding with the sky), whereas lower regions tend to contain more complex textures and chromatic variation (Igawa, Koga, Matsuzawa, & Nakamura, 2004). Because horizontal pixel shuffling preserves the vertical distribution of first-order chromatic information, these inherent asymmetries were present in both the natural-scene and pixel-shuffled conditions. It is therefore possible that part of the observed lower-field bias reflects the statistical structure of the stimuli themselves rather than, or in addition to, an asymmetry in visual field processing. Two observations show that some simple low-level accounts are insufficient on their own, although they do not exclude a low-level contribution in general, since pixel shuffling changed many image properties at once. First, if the preserved vertical chromaticity distribution alone was sufficient to produce the bias, a comparable effect should have appeared in the pixel-shuffled condition at 50 ms, which it did not (Experiment 2-1). Second, the luminance expansion effect, whereby high-luminance colors are perceived as larger (Sato, 1955; Vicovaro, Noventa, & Battaglini, 2019), would predict a bias toward the upper colored region (which typically corresponds with the brighter sky), which contradicts the observed pattern. These considerations suggest that the lower-field bias is not simply an artefact of stimulus-level asymmetries, although such influences cannot be entirely ruled out without additional controls, such as inverted natural-scene images.

The scope of the present conclusions is also limited by the use of only two stimulus types (natural-scene images and their pixel-shuffled counterparts). It remains unclear whether the observed lower-field bias generalizes to other types of visual content, such as paintings, faces, abstract art, or geometric patterns. Future research using a greater variety of stimuli would help to determine whether this effect is a general property of central color processing or whether it is selectively modulated by specific types of visual content.

Beyond these methodological considerations, our findings can also be situated within a broader evolutionary context. Our results are consistent with the broader idea that species-specific visual characteristics shaped by evolution may extend to processing biases in central vision. One such characteristic is the high cone density in the upper retina of humans, which supports the detailed processing of the lower visual field (Curcio et al., 1991). Just as the superior upper-field color vision of mice has been discussed in relation to the detection of aerial predators (Qiu et al., 2021; Szatko et al., 2020; Wallace et al., 2013), the human tendency to overevaluate lower-field color information may reflect an adaptive bias favoring the recognition of ground-level objects, food, and hazards. However, the present data do not directly test this evolutionary account, and such interpretations should be regarded as speculative.

## Limitations

This study has several limitations that should be addressed in future research.

First, as discussed in the General discussion, the pixel-shuffling manipulation did not isolate the semantic content from the other aspects of the image structure, and the residual low-level asymmetries were preserved in both conditions. Future studies should therefore include additional controls such as inverted natural-scene images, which would disrupt semantic content and typical spatial layout while creating a more symmetrical vertical chromaticity distribution.

Second, a standardized color vision test was not administered; instead, screening was based on self-report. Although this procedure was kept consistent across experiments, future studies should incorporate a formal color vision assessment. However, the expected impact of any undetected deficiency is small. The prevalence of red–green color vision deficiency in the Japanese population is approximately 5.0% in males and 0.2% in females (Birch, 2012). Given the predominantly male composition of our samples (16, 15, and 16 male participants in Experiments 1, 2-1, and 2-2, respectively), the expected number of participants with an undetected color vision deficiency is approximately one per experiment, with the contribution from female participants being negligible. Because color vision was not tested directly, however, the actual number cannot be known with certainty.

Third, the exclusion rate in Experiment 2-1 was notably higher than that in the other experiments: 6 of the 21 participants (28.6%) were excluded on the basis of the common exclusion criterion, compared with 0% in Experiment 1 (excluding one case of data corruption) and 0% in Experiment 2-2. This relatively high rate raises the possibility that the analyzed sample in Experiment 2-1 may not be fully representative of the general population. Specifically, if participants who were excluded differed systematically from those who were retained (e.g., in their sensitivity to the stimuli or in their response strategies), the results of Experiment 2-1 could overestimate or underestimate the true effect. One likely contributing factor is the short presentation duration (50 ms) in Experiment 2-1 combined with the inclusion of pixel-shuffled stimuli, which are more difficult to judge reliably in the absence of an intact scene structure. Experiment 2-2, which used an otherwise identical design but a longer duration (250 ms), excluded no participants under the same criterion, which is consistent with this interpretation. Future studies should consider strategies to reduce exclusion rates, such as additional practice trials or adaptive task designs, to ensure more representative samples. Importantly, a sensitivity analysis using a more lenient exclusion criterion (retaining approximately 80% of the sample) reproduced the key pattern of results (see Experiment 2-1 Analysis), indicating that the principal findings are not an artefact of the exclusion threshold.

## Conclusions

Taken together, the present findings show that the well-established lower-field advantage in color processing extends to central vision and is shaped by both the scene context and the processing time. The bias was strongest and emerged most rapidly when the natural-scene structure was preserved, and it extended to pixel-shuffled stimuli only when a longer processing time was available. A full mechanistic account will require direct measurement in future work, particularly of the proposed roles of attention and functional meaning.
